# A Preliminary Study on the Differentiation of Linseed and Poppy Oil Using Principal Component Analysis Methods Applied to Fiber Optics Reflectance Spectroscopy and Diffuse Reflectance Imaging Spectroscopy

**DOI:** 10.3390/s20247125

**Published:** 2020-12-12

**Authors:** Silvia Rita Amato, Aviva Burnstock, Anne Michelin

**Affiliations:** 1 The Courtauld Institute of Art, Somerset House, Strand, London WC2R 0RN, UK; 2 Centre de Recherche sur la Conservation, (CRC, USR 3224), Muséum National d’Histoire Naturelle, Ministère de la Culture et de la Communication, CNRS, 36 rue Geoffroy-Saint-Hilaire, CP21, 75005 Paris, France; anne.michelin@mnhn.fr

**Keywords:** paintings, artists’ paint media, drying oil, linseed oil, poppy oil, FORS, diffuse reflectance imaging spectroscopy

## Abstract

This paper presents results from the examination of a set of experimental samples using fibre optic reflectance spectroscopy (FORS) and diffuse reflectance imaging spectroscopy in the short-wave infrared (SWIR) range, combined with statistical analysis of the data for the discrimination and mapping of poppy and linseed oil. The aim was to evaluate the efficacy of this non-invasive approach for the study of paint samples with a view to the application of the method for characterisation of the two drying oils in painted art. The approach allowed discrimination between the two drying oils based on FORS spectra and the hyperspectral cube, indicating the influence of the spectral region around 1700 nm on the statistical discrimination based on the anti-symmetric and symmetric first overtone stretching of methylenic CH_2_ groups. This method is being studied as a potential non-invasive method of organic analysis of oil types that have formerly been studied using gas chromatography-mass spectrometry (GC-MS), which requires micro-samples.

## 1. Introduction

Linseed and poppy oil have been amongst the most common binding media used in oil painting. They are obtained from the seeds of the flax (*Linum usitatissimum*) and from seeds of the opium poppy (*Papaver somniferum*), respectively, and like the other vegetable drying oils, they are composed of mixed triglycerides of glycerol and fatty acids [1]. For linseed oil, the typical fatty acid composition is about 6–7% and 3–6% of the saturated palmitic acid and stearic acid, respectively; about 14–24% of the monounsaturated oleic acid; about 14–19% of the doubly unsaturated linoleic acid; and about 48–60% of the triply unsaturated linolenic acid. Poppy oil contains about 10% and 2% of the saturated palmitic acid and stearic acid, respectively; 11% of the monounsaturated oleic acid; about 72% of the doubly unsaturated linoleic acid; and 5% of the triply unsaturated linolenic acid [2]. Like the other vegetable drying oils, the drying process of linseed and poppy oil is due to polymerization reactions of the unsaturated fatty acids in the triglyceride molecules upon exposure to oxygen of the air [3]. Compared to poppy oil, linseed oil dries more quickly, forming a film that is harder and more durable, although it has a strong tendency to yellow, while the smaller content of linolenic acid in poppy oil makes this oil less prone to yellow, although this difference in composition also makes it more susceptible to cracking once dry [4].

Poppy oil was sometimes favoured over linseed oil to grind pigments, especially whites, blues, and greens, because of its paler colour and lower tendency to yellow, however it never replaced it as linseed oil was relatively fast drying and was recommended for poorly drying pigments [5]. Therefore, it is possible for both drying oils to be present in the same painting where they were used mixed with different pigments.

While differentiation of binding media in paintings has traditionally used chromatographic methods such as gas chromatography-mass spectrometry (GC-MS), or spectroscopic methods such as micro-Fourier transform infrared (µ-FTIR) spectroscopy, these techniques require micro-samples to be taken from the artworks. In recent years, there has been an increasing interest in the application of non-sampling, imaging methods for the identification and mapping of classes of organic painting materials [3,6,7,8,9,10,11]. Previous studies have shown the potential of reflectance imaging spectroscopy to identify and map different binding media using vibrational features associated with methylenic, CH, and amide functional groups, allowing for the characterisation and separation of different classes of organic materials in illuminated manuscripts, Early Renaissance, and modern paintings [9,10,11]. However, discrimination of organic compounds of the same class has proven to be more challenging; a range of methods of multivariate analysis have been coupled with spectroscopic techniques with the aim of extracting information from spectral differences that allow characterisation of different compounds from the same class [12,13,14,15].

This paper is part of an ongoing study of Impressionist painting techniques. Analyses of the binding media used by Impressionist artists based on gas-chromatography show the use of both linseed and poppy oil, and either walnut oil or mixtures of linseed and poppy oil [16,17,18,19]. This paper presents the results from the examination of a set of experimental samples using fibre optic reflectance spectroscopy (FORS) and diffuse reflectance imaging spectroscopy in the short-wave infrared (SWIR) range, processed using methods of statistical analysis for the discrimination and mapping of poppy and linseed oil. The aim was to evaluate the efficacy of this non-invasive approach to the study of simplified paint samples preliminary to the application of the method for characterisation of these two drying oils in painted art.

## 2. Materials and Methods

Experimental samples were prepared and examined using FORS spectroscopy and diffuse reflectance imaging spectroscopy in the SWIR range.

The canvas used for the samples was stretched, sized, and primed by hand using two simplified types of ground commonly found in Impressionist pictures: a lead white and chalk-based ground and a lead white and barium sulphate-based ground, which were created in-house [16].

Once the canvas was prepared, two dry pigments were individually hand-ground with linseed oil or poppy oil to make four types of paint that were applied onto both grounds and the bare canvas. The pigments selected were lead white and chrome yellow, a traditional white pigment and a modern yellow pigment, respectively, often present in the Impressionist palette and bound in poppy oil due to their light shade [16]. The aim was to explore the potential of the non-invasive approach used in this study to differentiate the two drying oils despite the use of different pigments and different grounds.

**Figure 1:**
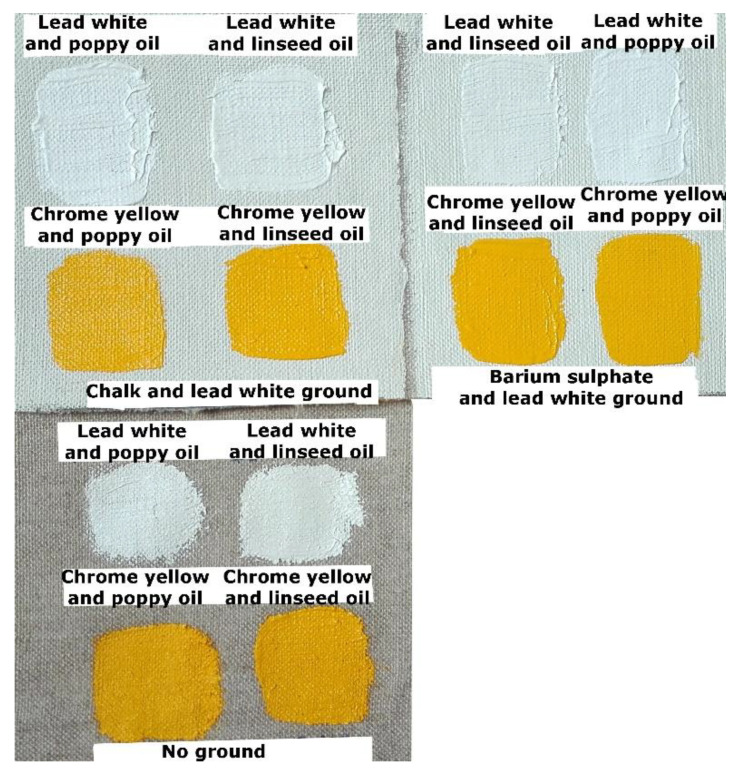
Normal light image of the experimental samples examined.

The samples were allowed to cure and naturally age in ambient indoor conditions for about 40 days. Details of the materials—and their suppliers—used to produce the samples are given in Table 1, and an image of the samples is given in Figure 1.

### 2.1. Fibre Optics Reflectance Spectroscopy

**Figure 2:**
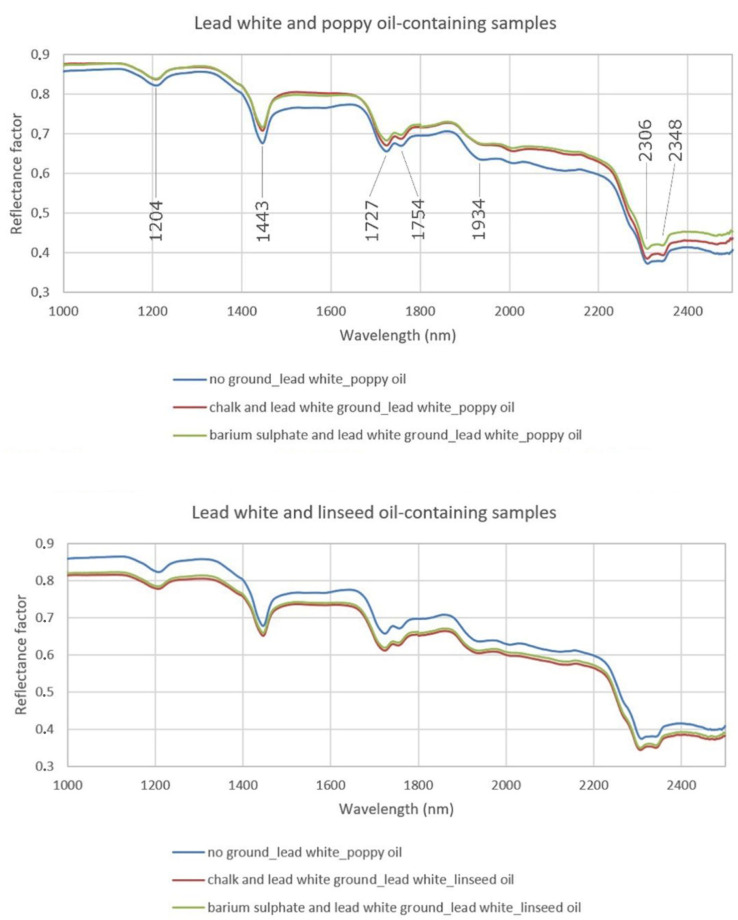

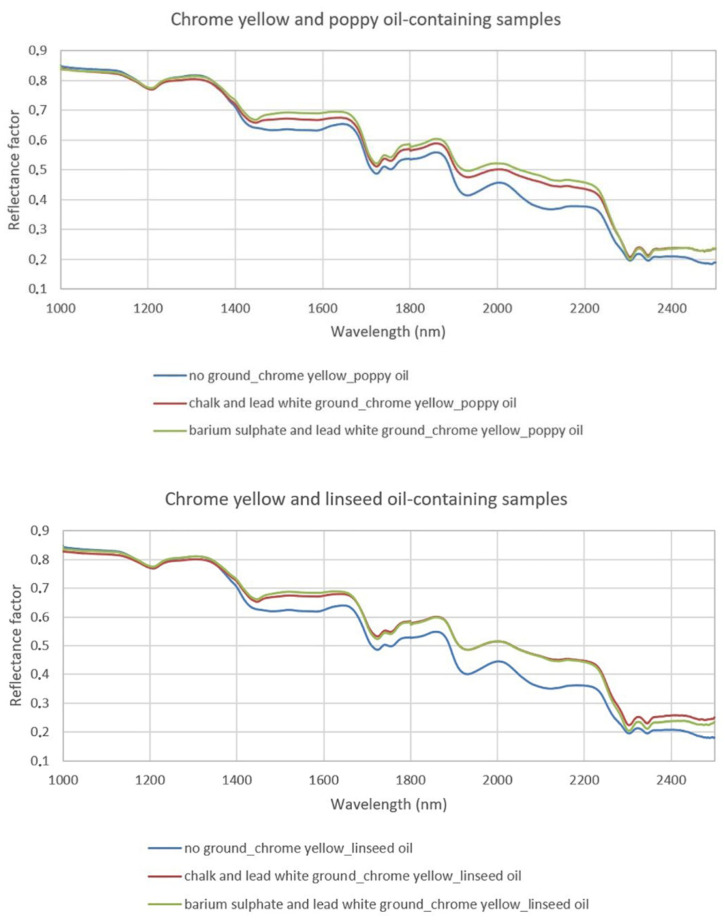
Fibre optic reflectance spectroscopy (FORS) spectra acquired from the different experimental samples.

**Figure 3:**
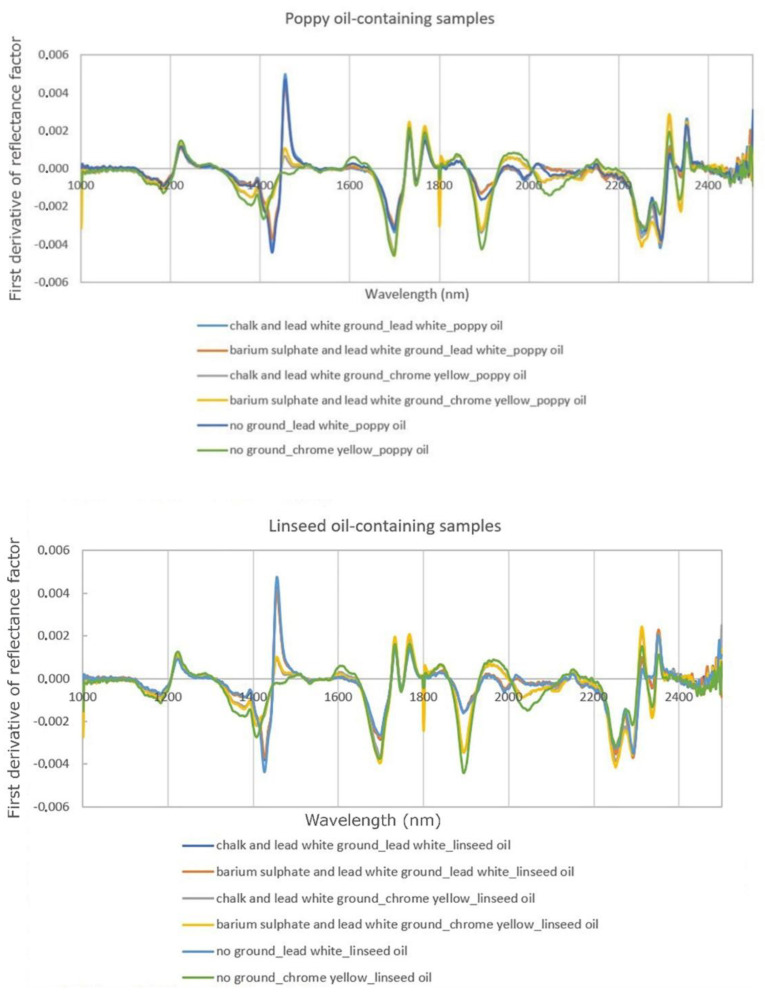
First derivative of the FORS spectra acquired from the experimental samples.

Fibre optic reflectance spectroscopy (FORS) was performed using a portable fibre spectrophotometer FieldSpec 4 (“Hi-Resolution”, ASD Inc.., Boulder, Colorado, United States) equipped with a visible to near-infrared(VNIR) detector (350–1000 nm) consisting of a 512-element silicon array and two short-wave infrared radiation (SWIR) detectors (1000–1800 nm and 1800–2500 nm) consisting of Graded Index InGaAs Photodiodes, Two Stage TE Cooled. The spectral resolution was 3 nm in the range 350–1000 nm and 8 nm in the range 1000–2500 nm. The system was coupled with a bundle of quartz-silica optical fibres transmitting in the 350–2500 nm spectral region connected to a contact probe-head operating in the 0°/45° geometry, which directed the halogen light source (colour temperature 2900 K) to the surface of the sample and allowed for capturing the light reflected from an area of about 3 mm^2^. Calibration was performed using a Spectralon^®^ (Labsphere) tile. Five spectra were acquired for each paint sample. The average spectra were calculated for each paint sample and the results are shown in Figure 2. The spectral range investigated in this study for the FORS spectra was 1000–2500 nm. The first-derivative transformation of the reflectance spectra was made using OriginPro 8.5 with a Savitzky–Golay algorithm. The average first-derivative transformation was calculated for each group of reflectance spectra and the results are given in Figure 3. Principal component analysis (PCA) of the first-derivative transformation of all the reflectance spectra acquired from each paint sample was performed with R [20].

The different groups of samples in the PCA were labelled “BASLW(or CLW)_CY(or LW)_LO (or PO)”, where BASLW (or CLW) stands for barium sulphate and lead white ground (or, in the case of CLW, chalk and lead white ground); CY (or LW) identifies the pigment used in each paint (chrome yellow or lead white); and LO (or PO) identifies the binding medium (linseed oil or poppy oil) used in each paint. The samples from the area of the canvas where no ground layers were present were labelled as CY (or LW)_LO (or PO).

### 2.2. Diffuse Reflectance Imaging Spectroscopy

Hyperspectral imaging of the samples in the SWIR spectral range was carried out using an ImSpector N25E 2/3” spectrograph purchased from Specim Corp. (Finland). This is a push-broom imaging device coupled with a Peltier-cooled MCT detector (9.6 mm detector having 320 (spatial) × 256 (spectral) pixels) operating in the 1000–2500 nm range, with 10 nm spectral resolution. The fore objective lens used was OLES15 (focal length 1/4 15 mm). The light source was composed of six halogen lamps (35W) positioned on the two sides of the detector and the camera was calibrated with white reference (99% reflectance–Spectralon^®^). The first derivative of the reflectance cube with respect to wavelength and the minimum noise fraction transform (MNF) of the data were performed with the ENVI software (ENVI 5.5, Harris Geospatial).

## 3. Results and Discussion

The FORS spectra collected from the paint samples are shown in Figure 2. While chrome yellow has no distinctive bands in the SWIR range, the spectra collected from the lead white-containing samples showed the OH stretching overtone and the carbonate anion combination bands at about 1443 and 2320 nm respectively characteristic of hydrocerussite [21]. All the acquired spectra, five for each paint sample, exhibited SWIR spectral features characteristic of drying oils from the lipid triglyceride molecules they contain, in agreement with the bands assigned by Vagnini et al. [7]. These are highlighted in Figure 2. The bands that occur as a doublet at about 1727 and 1754 nm are due to the first antisymmetric/symmetric CH_2_ stretching overtone, while the band at 1204 nm is due to the second CH_2_ stretching overtone. At 1934 nm, a band related to the first overtone of the ester carbonyl stretching mode could be noted, while the doublet near 2306 and 2348 nm is related to the antisymmetric/symmetric stretching and bending of the methylenic CH_2_ groups.

In agreement with previous research by other authors, no significant spectral differences between the paints containing poppy oil and those containing linseed oil were detectable in the reflectance spectra [7,14]. Thus, the first derivative with respect to wavelength (Figure 3) was calculated for all the spectra with the aim of investigating potential differences that might arise and be used for separating and mapping the two kinds of oil used as paint binding media. First derivative can be a useful tool to differentiate key spectral features as it eliminates intensity differences in the reflectance spectra, provides enhanced resolution and discrimination in favour of the sharpest features of a spectrum, and reduces the interferences from broadband constituents [22]. This approach has been used successfully in previous studies that employed spectral imaging to discriminate and map different binding media in Early Renaissance and modern paintings [10,11].

**Figure 4:**
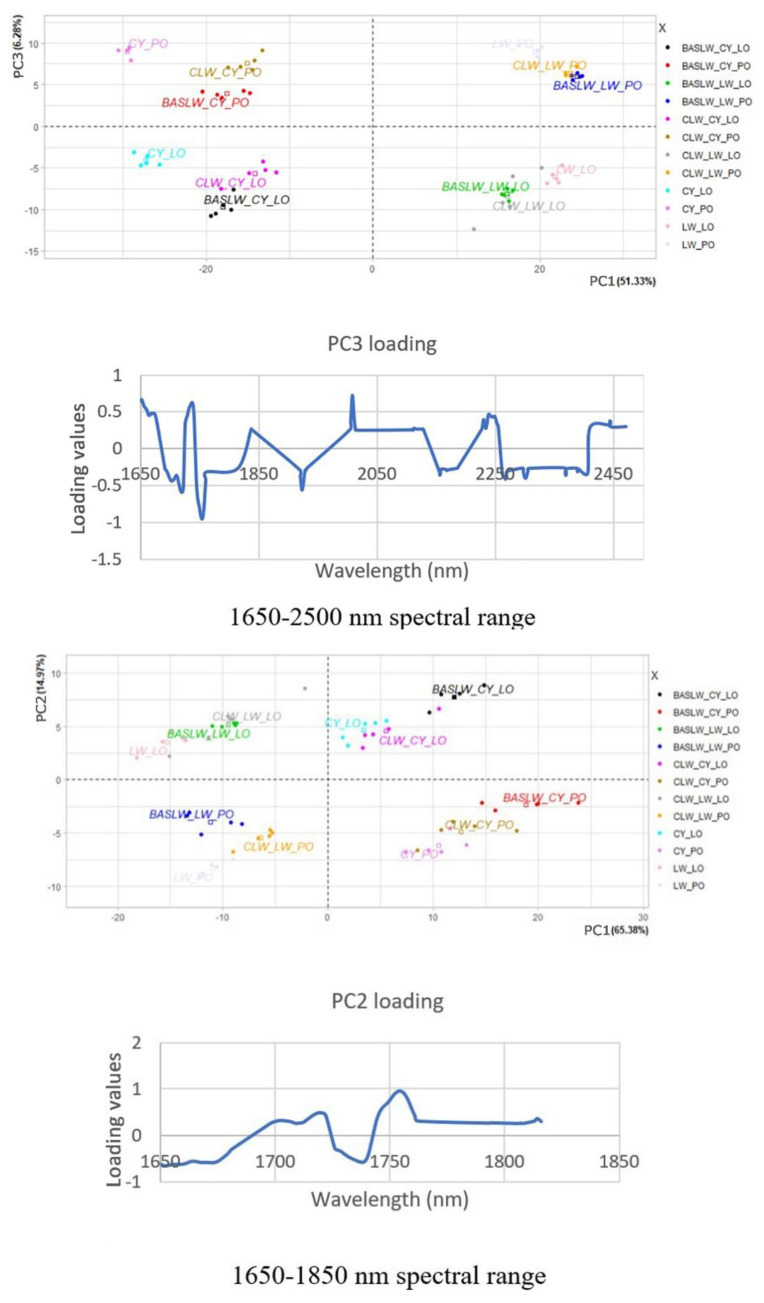

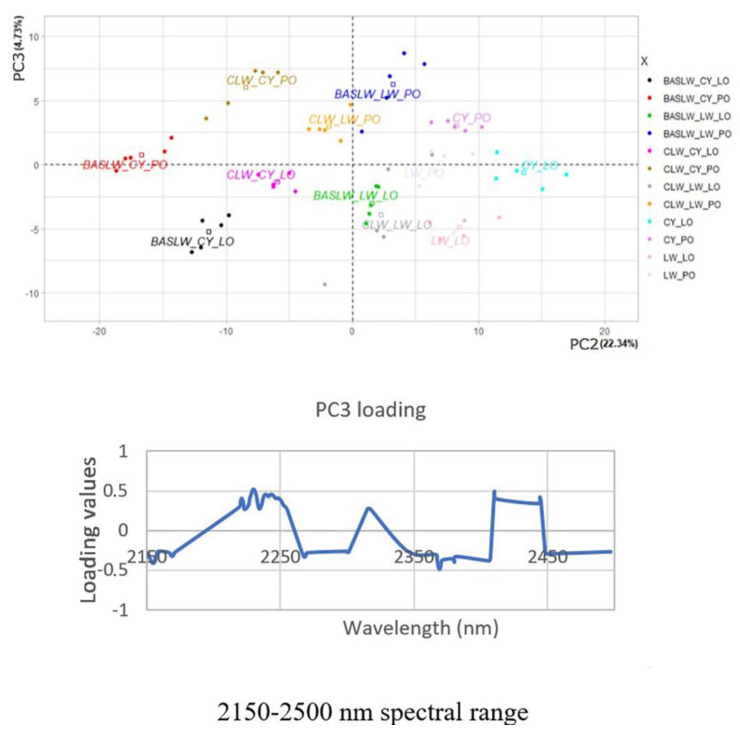
Score plots and related loading plots of principal component analysis (PCA) applied to the first derivative of the FORS spectra acquired from the experimental samples. The different groups of samples are labelled “BASLW(or CLW)_CY(or LW)_LO (or PO)”, where BASLW (or CLW) stands for barium sulphate and lead white ground (or, in the case of CLW, chalk and lead white ground); CY (or LW) identifies the pigment used in each paint (chrome yellow or lead white); LO (or PO) identifies the binding medium (linseed oil or poppy oil) used in each paint. The samples from the area of the canvas where no ground layers were present are labelled as CY (or LW)_LO (or PO).

The derivative spectra of the paints containing poppy oil and those containing linseed oil are closely similar to each other, distinguished only by variations in the intensity of the peaks (Figure 3). In order to facilitate the data exploration and to simplify the classification of the spectra, principal component analysis was applied to the derivative spectra in the ranges 1650 to 2500 nm, 1650 to 1850 nm, and 2150 to 2500 nm (Figure 4), corresponding to ranges where absorption bands of the two drying oils occur. It was decided not to include the spectral region around 1443 nm, corresponding to the absorption band for lead white, as not all paint samples contained only lead white. PCA was also performed on derivative spectra in the same spectral ranges, but for smaller datasets (only chrome yellow containing paints applied on both ground layers; only lead white paints applied on both ground layers; both chrome yellow and lead white paints applied on both ground layers; both chrome yellow and lead white paints applied on unprimed canvas. See Figures S1–S3 in Supplementary Materials).

The aim was to investigate the potential of PCA to highlight spectral features that might have an impact in the differentiation of the two drying oils and to investigate whether the pigments and the ground layers have an influence on the results.

A selection of results from the PCA of the samples is illustrated in Figure 4. For each spectral range investigated, the score plot of the principal component that separates more clearly linseed oil containing samples from those containing poppy oil is included, followed by the related loading plot that shows how strongly each spectral band influences that principal component. For each dataset, the resulting bands were organised by decreasing loading value in Table 2. The results show that the largest variability of the different sets of data can be explained by the first two to three PCs. Although no characteristic bands related to the pigments used were detected in the spectral ranges investigated in this study, generally, the first PC contributes to the discrimination between the two pigments used, while the second PC contributes to the discrimination of the samples containing the two drying oils. When paint samples containing only one pigment are included in the dataset, generally the first PC contributes to the discrimination of the samples containing the two drying oils (see Figures S1–S3 in Supplementary Materials). In the case of real artworks, the presence of different pigments in complex paint mixtures might interfere and make discrimination of the two drying oils more difficult. However, in the case of this study, discrimination was possible, regardless of the pigments used and spectral range considered.

PCA allowed differentiation between the samples containing the two different drying oils in almost all cases. Overall, the data showed that the greatest contributions to the PCs that discriminated between the two drying oils were due to the spectral regions around 1724, 1738, and 1754 nm (see Table 2 and also Table S1 in the Supplementary Materials). The signals around 1724 and 1754 nm always had opposite values (+/−) compared to the signal at around 1738 nm, indicating that the first two were directly correlated. Examination of the reflectance spectra of the samples and of their first derivatives showed that the signals at 1724 nm and at 1754 nm appeared as peaks in the reflectance spectra and as zero crossing points in their first derivative. The signal at 1738 nm was a minimum in the reflectance spectrum and a zero-crossing point in the first derivative. When the smaller range of 2150–2500 nm was considered, it was still possible to discriminate between the samples containing the two different oils. As illustrated below, the spectral variations in this range are likely due to contributions from the antisymmetric/symmetric stretching and bending of the methylenic CH_2_ groups. However, discrimination in the range of 2150–2500 nm was less distinctive, suggesting that this spectral range is less discriminating than the range around 1700 nm. This is due to the influence of spectral features from both the pigments and other materials used to prepare the samples. Examples are the carbonate anion combination band of hydrocerussite in the 2300 nm region present in the spectra collected from the lead white-containing samples, which alter the doublet near 2306 and 2348 nm related to the antisymmetric/symmetric stretching and bending of the methylenic CH_2_ groups in the drying oils, and the absorption band at about 2100 nm due to the combination of OH and CH stretching vibrations characteristic of cellulose in the canvas, which is present in the spectra collected from the paint samples where chrome yellow paint was applied onto the unprimed canvas, while it is not visible in those spectra collected from paint samples where lead white is present in the ground and in the paint layers [23].

The samples were more loosely grouped in the PCs when the ground layers were present (see Figures S1–S3 in Supplementary Materials). However, in the latter case, discrimination between the samples containing the two drying oils was still possible, suggesting that the ground layers have a minor influence on the results.

In drying oils, anti-symmetric and symmetric first overtone stretching of methylenic C–H are at about 1724 and 1754 nm, and the results from the PCA analysis suggests that the spectral regions around these signals are likely to play a role in the discrimination between paint samples containing linseed oil and those containing poppy oil. A study by Carlesi et al. on model samples of pure binders showed that different drying oils could be differentiated based on Raman and Fourier transform-near-infrared (FT-NIR) first derivative spectra processed using PCA, and that good statistical discrimination could be achieved based on the contributions from the spectral regions of the ν(C = C) fundamental stretching (1700–1600 cm^−1^ or 5880–6250 nm) and of the methylenic stretching and bending combination bands in the range of 4800–4200 cm^−1^ (2080–2380 nm) [14]. The authors hypothesised that the different contributions of the methylenic stretching and bending bands might be linked to those methylene groups located between nonconjugated bonds that are involved in the autoxidation reactions within the drying process of the oils. Thus, it has been hypothesised that their contributions, together with those from the C=C stretching, are likely to be related to the different polyunsaturated fatty acid contents of the different drying oils [3,7,8,9]. In a similar way, it could be hypothesised that the spectral regions around the anti-symmetric and symmetric first overtone stretching of methylenic CH_2_ at about 1724 and 1754 nm highlighted by PCA analysis in the present study could also be linked to the different polyunsaturated fatty acid contents of the different drying oils, allowing for their differentiation with PCA.

A similar approach was used to treat the hyperspectral data from the SWIR cube of the samples. The reflectance cube was first transformed into a first derivative (with respect to wavelength) cube to which PCA was applied. The two drying oils in the different paints were not satisfactorily differentiated and mapped. Thus, given that the hyperspectral data produced in this study had a lower signal-to-noise ratio compared to the FORS spectra illustrated above, it was chosen to apply a minimum noise fraction transform (MNF) to the first derivative cube. Both PCA and MNF are multivariate methods for the reduction of spectral bands. PCA reorganises the variance of a hyperspectral cube into a new coordinate system, of which the axes, or principal components (PCs), are specific uncorrelated linear combinations of the original dataset. The variance is maximised and contained for the largest percentage within the first few PC image bands, which thus contain most of the coherent image information. Like PCA, MNF also transforms the original dataset into new components that are ordered by image quality, although in this case, the new components are chosen to maximise the signal-to-noise ratio instead of the variance [10,24].

**Figure 5:**
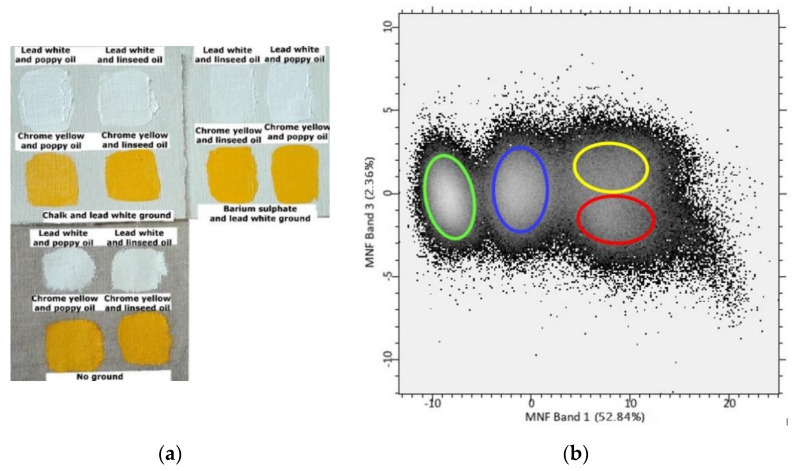

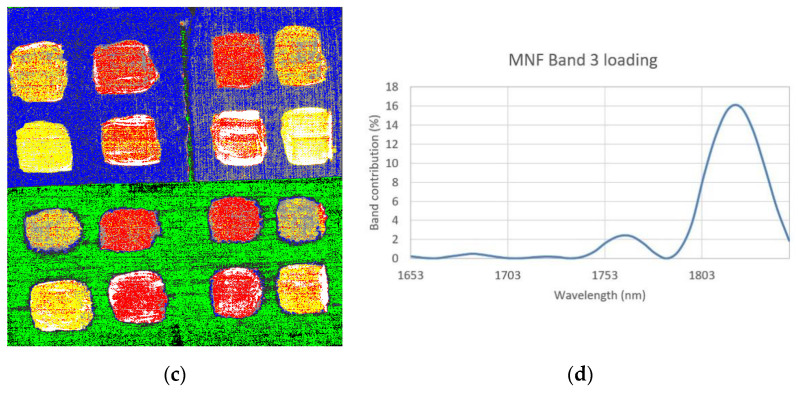
(**a**) Visible light image of the samples; (**b**) scatter plot of minimum noise fraction transform (MNF) band 1–band 3. Range 1653–1848 nm. The different classes identified in the scatter plot have been highlighted by hand using different colours and their distribution in the samples is visualized in the classified image; (**c**) classified image based on the classes identified in the scatter plot; (**d**) loading plot of MNF band 3.

Given that the PCA of the FORS data showed that the greatest contributions for the discrimination between linseed oil and poppy oil were due to the spectral regions around 1724, 1738, and 1754 nm, MNF of the first derivative of the SWIR hyperspectral cube was applied only in the spectral range from 1653 to 1848 nm. The results are shown in Figure 5 (see Figures S4–S7 in Supplementary Materials for the results from the smaller datasets). As illustrated by the scatter plot in Figure 5b, MNF band 3 contributed to the differentiation between the samples containing the two different drying oils in this spectral range (see the classified image of the samples given in Figure 5c). The different classes identified in the scatter plot (Figure 5b) have been highlighted by hand using different colours and their distribution in the samples is visualized in the classified image (Figure 5c). The yellow class refers to the poppy oil-containing samples, the red class refers to the linseed oil-containing samples, while the blue and the green classes refer to the primed canvas and the unprimed canvas, respectively. However, the separation between the groups of samples in the scatter plot obtained was less clearly defined than in the scatter plots obtained with the PCA of the FORS data.

The wavelengths that more strongly influence MNF band 3 and that more clearly separate linseed oil containing samples from those containing poppy oil are shown in the loading plot given in Figure 5d and are summarised in Table 2 (see also Table S2 in Supplementary Materials for the different datasets). Although not corresponding completely to the results from the PCA carried out on the first derivative of the FORS spectra, the results from the MNF analysis of the first derivative of the SWIR hyperspectral cube showed that the signals in the range of 1720–1780 nm contributed greatly to the discrimination of the two oils. This result is in agreement with the findings of the PCA applied to the FORS data, supporting the hypothesis discussed above that the spectral range of the anti-symmetric and symmetric first overtone stretching of methylenic CH_2_ plays a role in the differentiation of the two oils.

The difference in the results of the PCA of the FORS spectra, and MNF of the hyperspectral cube is likely to be due to the higher spectral resolution and better signal-to-noise ratio of the fibre optics spectrometer used to collect the FORS spectra compared to the hyperspectral camera used to acquire the hyperspectral cube of the samples.

## 4. Conclusions

The results of the analysis of the experimental paint samples produced using linseed oil and poppy oil showed that the application of methods of statistical analysis to FORS and diffuse reflectance imaging spectroscopy data in the SWIR range allowed discrimination between the two drying oils used. In the case of the FORS spectra, the results indicated good separation between the groups of samples containing the two oils and the influence of the spectral regions around 1724, 1738, and 1754 nm on the statistical discrimination. It has been hypothesised that these contributions might be related to the anti-symmetric and symmetric first overtone stretching of methylenic CH_2_ at about 1724 and 1754 nm of the two oils, and that they might be linked to the different polyunsaturated fatty acid contents of the two drying oils.

Differentiation between the oil types was also possible from statistical analysis of the diffuse reflectance imaging data in the SWIR range, although in this case, the separation between the groups of samples was less clear-cut. The results did not correspond completely to those obtained from the statistical analysis of the FORS spectra. However, the findings showed a strong contribution of the signals in the 1720–1780 nm range to the discrimination of the two oils, indicating a partial agreement with the results from the statistical analysis of the FORS spectra.

It was concluded that the difference in terms of results between the PCA of the FORS spectra and MNF of the hyperspectral cube was probably due to the higher spectral resolution and better signal-to-noise ratio of the fibre optics spectrometer used to collect the FORS spectra compared to the hyperspectral camera used to acquire the hyperspectral cube of the samples.

The results obtained in this study provided data that can be used to differentiate and map linseed and poppy oil used in experimental samples of oil paints. Further research will be required to explore the way the spectral range 1724–1754 nm contributes to the differentiation of these two types of drying oils, which could include, for example, examining the ratio between the bands or comparing the area under the curve of these bands.

The analytical approach used in this study on experimental samples will need to be tested on real artworks to evaluate and assess its applicability where more complex mixtures of pigments, organic media, varnish coatings, and later materials associated with the conservation of the works are present, since the overlapping of spectral features from different materials in the same spectral range may represent a limitation to the characterisation of the materials. Further research would also include examination of paint samples containing linseed and poppy oil mixtures, linseed and poppy oil from different suppliers, and a study on the potential influence of aging of these drying oils on the analytical results.

## Figures and Tables

**Table 1:** Materials used to produce the experimental samples and suppliers of the materials.

Unprimed canvas—linen cotton canvas 77 in/196 cm 305 gsm Size—Brodie & Middleton rabbit skin glue size Winsor & Newton cold pressed linseed oil Roberson poppy oil Barium sulphate—Kremer, dry pigment Chalk—calcium carbonate, Kremer, dry pigment Chrome yellow—Cornellissen & Son, dry pigment Lead white—Cornellissen & Son, dry pigment

**Table 2:** Bands that have more impact on the principal components illustrated in Figure 4 and Figure 5, organised by decreasing loading value.

Dataset	1650–2500 nm	1650–1850 nm	2150–2500 nm
FORS spectra	1753 nm (−), 2009 nm (+), 1719 nm (−), 1738 nm (+), 1923 nm (−), 1674 nm (+), 2242 nm (+), 2267 nm (−), 2301 nm (−)	1754 nm (+), 1738 nm (−), 1652 nm (−), 1669–1673 nm (−), 1720 nm (+)	2230 nm (+), 2369 nm (−), 2410 nm (+), 2240 nm (+), 2244 nm (+), 2249 nm (+), 2444 nm (+), 2155 nm (−), 2221 nm (+), 2268 nm (−), 2169 nm (−), 2300 nm (−), 2318 nm (+)
Hyperspectral cube	-	1820 nm, 1760 nm, 1685 nm, 1720 nm	-

## Supplementary Materials

The following are available online at https://www.mdpi.com/1424-8220/20/24/7125/s1, Figure S1: Score plots and related loading plots of PCA applied to the first derivative of the FORS spectra acquired from the experimental samples in the range 1650–2500 nm. Figure S2: Score plots and related loading plots of PCA applied to the first derivative of the FORS spectra acquired from the experimental samples in the range 1650–1850 nm. Figure S3: Score plots and related loading plots of PCA applied to the first derivative of the FORS spectra acquired from the experimental samples in the range 2150–2500 nm. Figure S4: Top left: visible light image of the samples. Top right: scatter plot of MNF band 1–MNF band 2 of chrome yellow paints applied on both ground layers. Range 1653–1848 nm. Bottom left: classified image based on the classes identified in the scatter plot. Bottom right: loading plot of MNF band 2. Figure S5: Top left: visible light image of the samples. Top right: scatter plot of MNF band 1–MNF band 2 of lead white paints applied on both ground layers. Range 1653–1848 nm. Bottom left: classified image based on the classes identified in the scatter plot. Bottom right: loading plot of MNF band 2. Figure S6: Top left: visible light image of the samples. Top right: scatter plot of MNF band 1–MNF band 3 of lead white and chrome yellow paints applied on both ground layers. Range 1653–1848 nm. Bottom left: classified image based on the classes identified in the scatter plot. Bottom right: loading plot of MNF band 3. Figure S7: Top left: visible light image of the samples. Top right: scatter plot of MNF band 1–MNF band 2 of lead white and chrome yellow paints applied on the bare canvas. Range 1653–1848 nm. Range 1653–1848 nm. Bottom left: classified image based on the classes identified in the scatter plot. Bottom right: loading plot of MNF band 2. Table S1: Bands that have more impact on the principal components illustrated in Figures S1 to S3, organised by decreasing loading value. Table S2: Bands that have more impact on the MNF bands illustrated in Figures S4 to S7, organised by decreasing loading value.
