# Supplementary material for: A Preliminary Study on the Differentiation of Linseed and Poppy Oil Using Principal Component Analysis Methods Applied to Fiber Optics Reflectance Spectroscopy and Diffuse Reflectance Imaging Spectroscopy
Source: Sensors (Basel). 2020 Dec 12;20(24):7125. doi: 10.3390/s20247125 (PMC7764422; doi:10.3390/s20247125)
Supplement: Supplementary file 1 [file sensors-20-07125-s001.zip › Table S1.docx]

| Samples | 1650-2500 nm | 1650-1850 nm | 2150-2500 nm |
| --- | --- | --- | --- |
| Chrome yellow paints applied on both ground layers | 1730-1738 nm (+), 2268 nm (-), 2317 nm (+), 1700-1717 nm (-), 1747-1752 nm (-), 2340 nm (-), 2296-2301 nm (-), 1786 nm (+), 1909 nm (+), 2354 nm (+) | 1768-1780 nm (+), 1695-1720 nm (-), 1730-1740 nm (+), 1746-1751 nm (-) | 2239 nm (+), 2247 nm (+), 2154 nm (-), 2230 nm (+), 2305 nm (-), 2169 nm (-), 2161 nm (-), 2370 nm (-), 2181 nm (-), 2450 nm (-), 2176 nm (-), 2217 nm (-), 2222 nm (+), 2357 nm (+), 2289 nm (+) |
| Lead white paints applied on both ground layers | 2300-2305 nm (+), 2039 nm (-), 1753 nm (+), 1903 nm (-), 1721 nm (+), 1949 nm (+), 1738 nm (-), 2179 nm (+), 2354 nm (-), 2317 nm (-), 1673 nm (-), 2330 nm (-), 2270 nm (+) | 1682 nm (+), 1762 nm (-), 1724 nm (-), 1683 nm (+), 1668 nm (+) | 2301 nm (+), 2354 nm (-), 2317 nm (-), 2175 nm (+), 2330 nm (-), 2269 nm (+), 2249 nm (-), 2243 nm (-), 2345 nm (+), 2405 nm (+), 2367 nm (+), 2411 nm (-) |
| Chrome yellow and lead white paints applied on both ground layers | 1752 nm (+), 1739 nm (-), 2143 nm (+), 2008 nm (-), 2264 nm (+), 2354 nm (-), 1722 nm (+), 2367 nm (+), 1924 nm (+) | 1754 nm (+), 1740 nm (-), 1673 nm (-), 1723 nm (+) | 2263 nm (+), 2352 nm (-), 2284 nm (+), 2291 nm (+), 2225 nm (+), 2166 nm (+), 2187 nm (+), 2218 nm (+), 2373 nm (+), 2415 nm (-), 2473 nm (-), 2441 nm (-), 2317 nm (-) |
| Chrome yellow and lead white paints applied on bare canvas | 1738 nm (+), 1754 nm (-), 2266 nm (-), 1923 nm (-), 1657 nm (+), 2008 nm (+), 1719 nm (-), 2249 nm (+), 2114 nm (+) | 1756 nm (-), 1739 nm (+), 1657 nm (+), 1721 nm (-) | 2266 nm (+), 2276 nm (+), 2443 nm (-), 2422 nm (-) 2401 nm (+), 2437 nm (+), 2449 nm (+), 2396 nm (-) |
