# Supplementary material for: A Preliminary Study on the Differentiation of Linseed and Poppy Oil Using Principal Component Analysis Methods Applied to Fiber Optics Reflectance Spectroscopy and Diffuse Reflectance Imaging Spectroscopy
Source: Sensors (Basel). 2020 Dec 12;20(24):7125. doi: 10.3390/s20247125 (PMC7764422; doi:10.3390/s20247125)
Supplement: Supplementary file 1 [file sensors-20-07125-s001.zip › Table S2.docx]

| Samples | 1653-1848 nm |
| --- | --- |
| Chrome yellow paints applied on both ground layers | 1816 nm |
| Lead white paints applied on both ground layers | 1728 nm, 1779-85 nm, 1672 nm |
| Chrome yellow and lead white paints applied on both ground layers | 1728 nm, 1666 nm, 1785 nm, 1835 nm |
| Chrome yellow and lead white paints applied on bare canvas | 1728 nm, 1672 nm, 1779 nm |
