# Supplementary figures and images for: A Preliminary Study on the Differentiation of Linseed and Poppy Oil Using Principal Component Analysis Methods Applied to Fiber Optics Reflectance Spectroscopy and Diffuse Reflectance Imaging Spectroscopy
Source: Sensors (Basel). 2020 Dec 12;20(24):7125. doi: 10.3390/s20247125 (PMC7764422; doi:10.3390/s20247125)

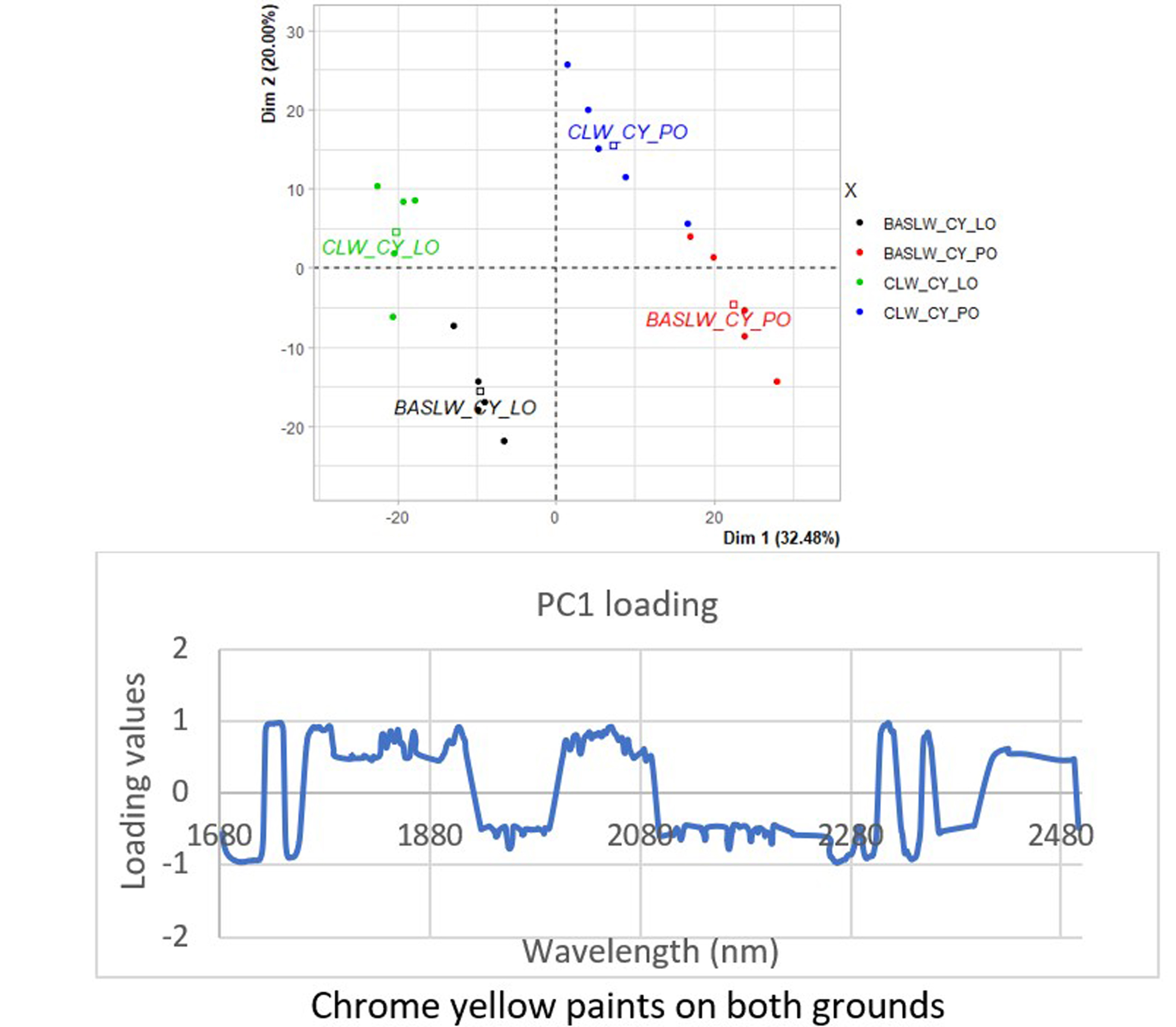

Supplement: Supplementary file 1 [file sensors-20-07125-s001.zip › Figure S1_1.jpg]

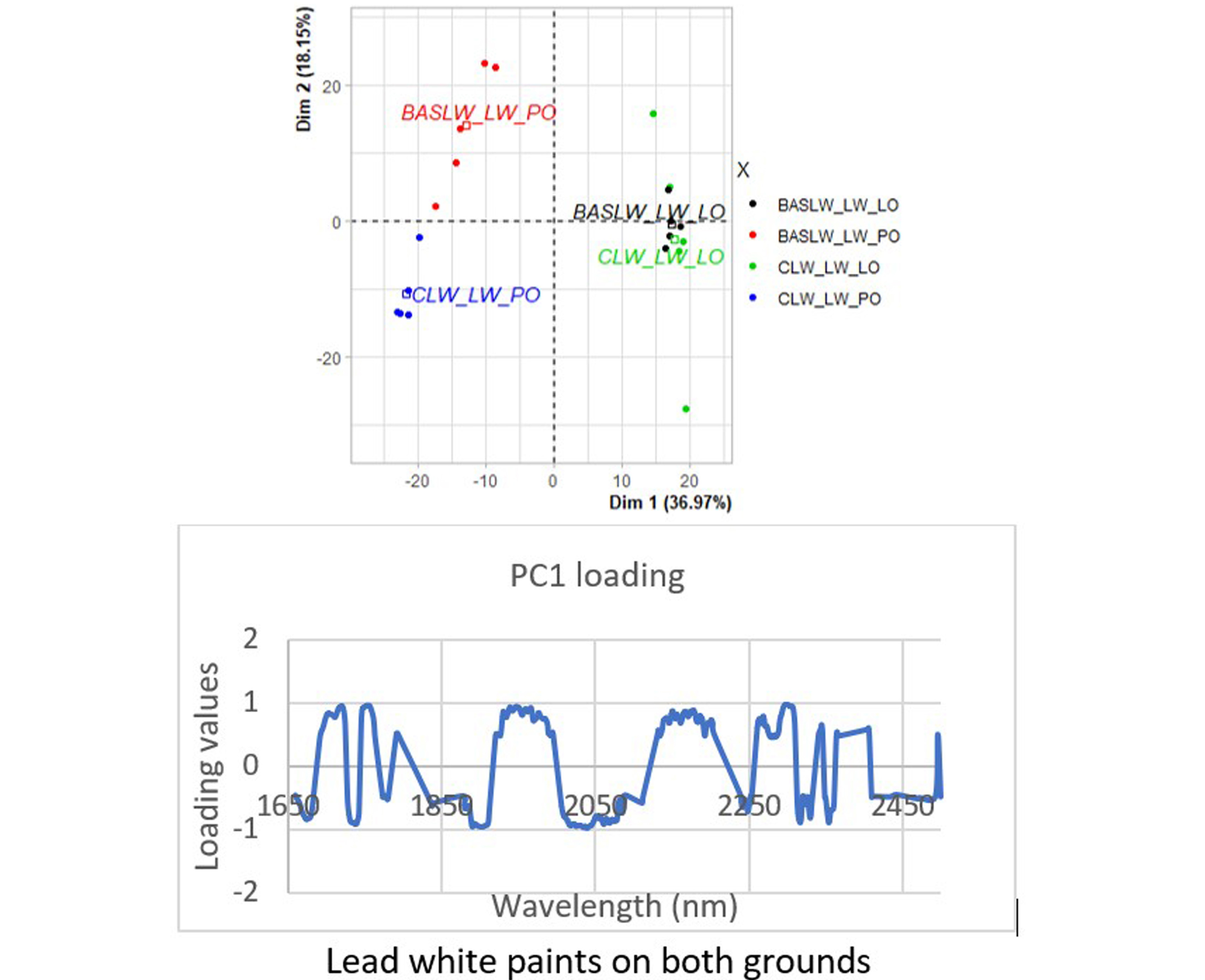

Supplement: Supplementary file 1 [file sensors-20-07125-s001.zip › Figure S1_2.jpg]

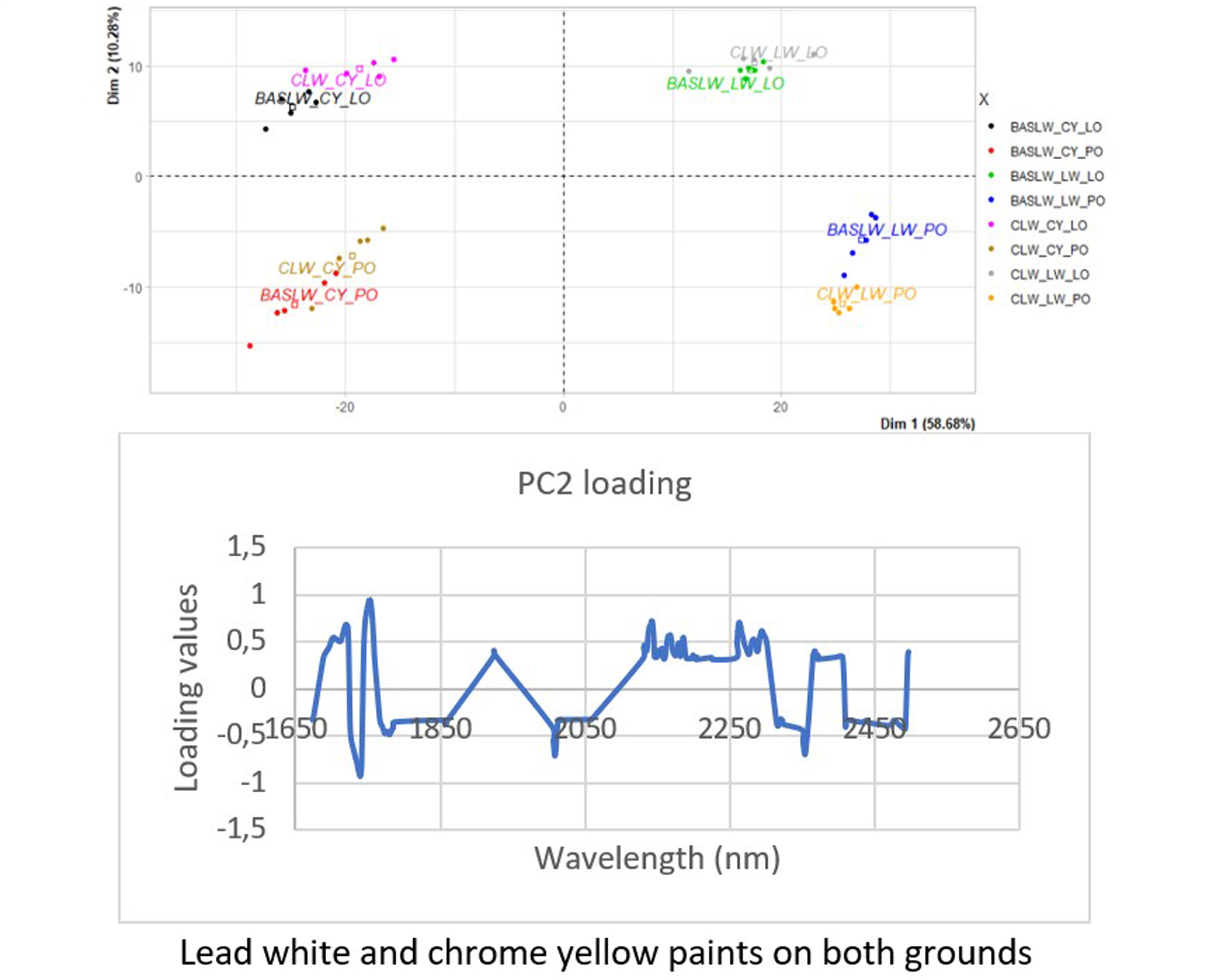

Supplement: Supplementary file 1 [file sensors-20-07125-s001.zip › Figure S1_3.jpg]

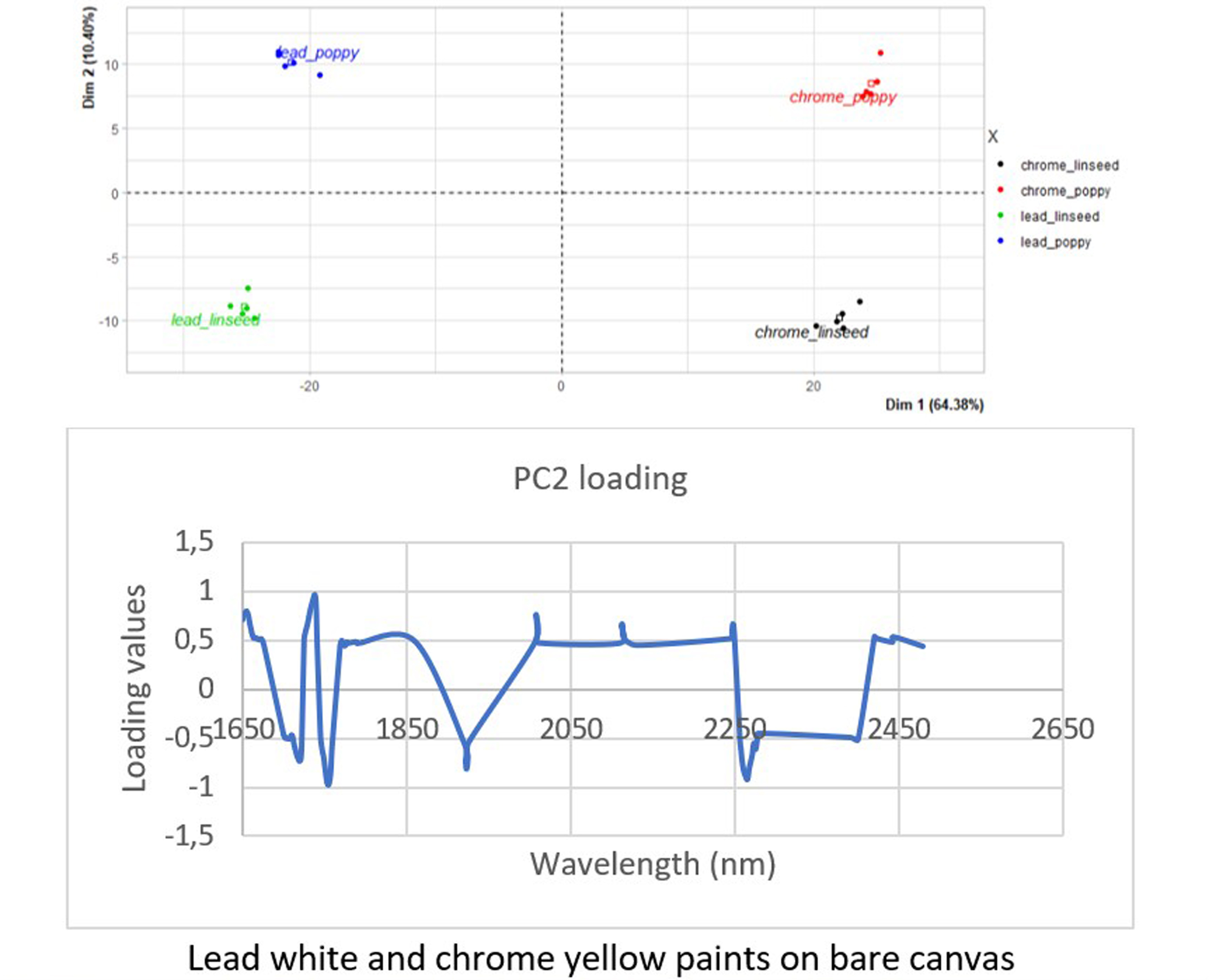

Supplement: Supplementary file 1 [file sensors-20-07125-s001.zip › Figure S1_4.jpg]

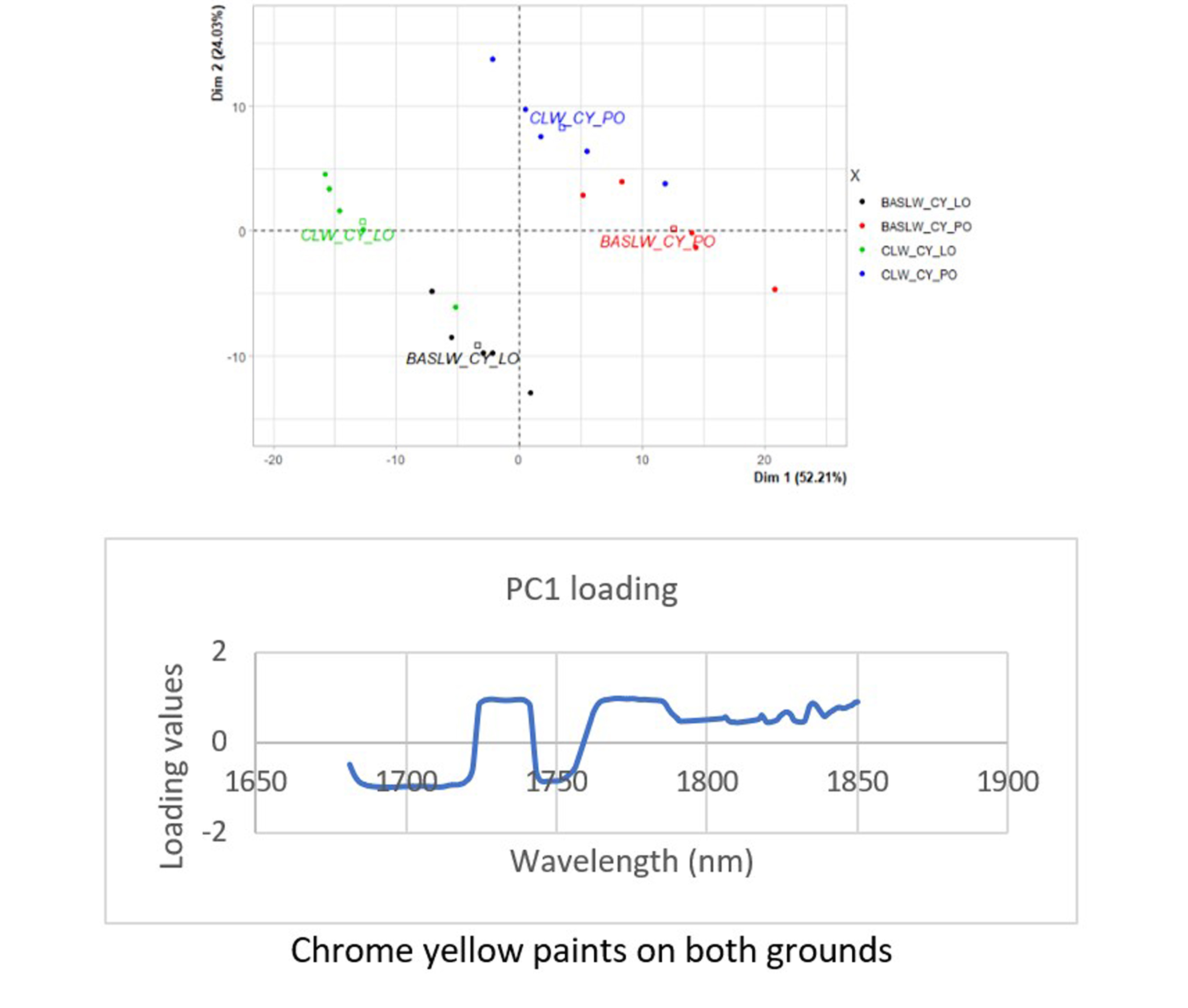

Supplement: Supplementary file 1 [file sensors-20-07125-s001.zip › Figure S2_1.jpg]

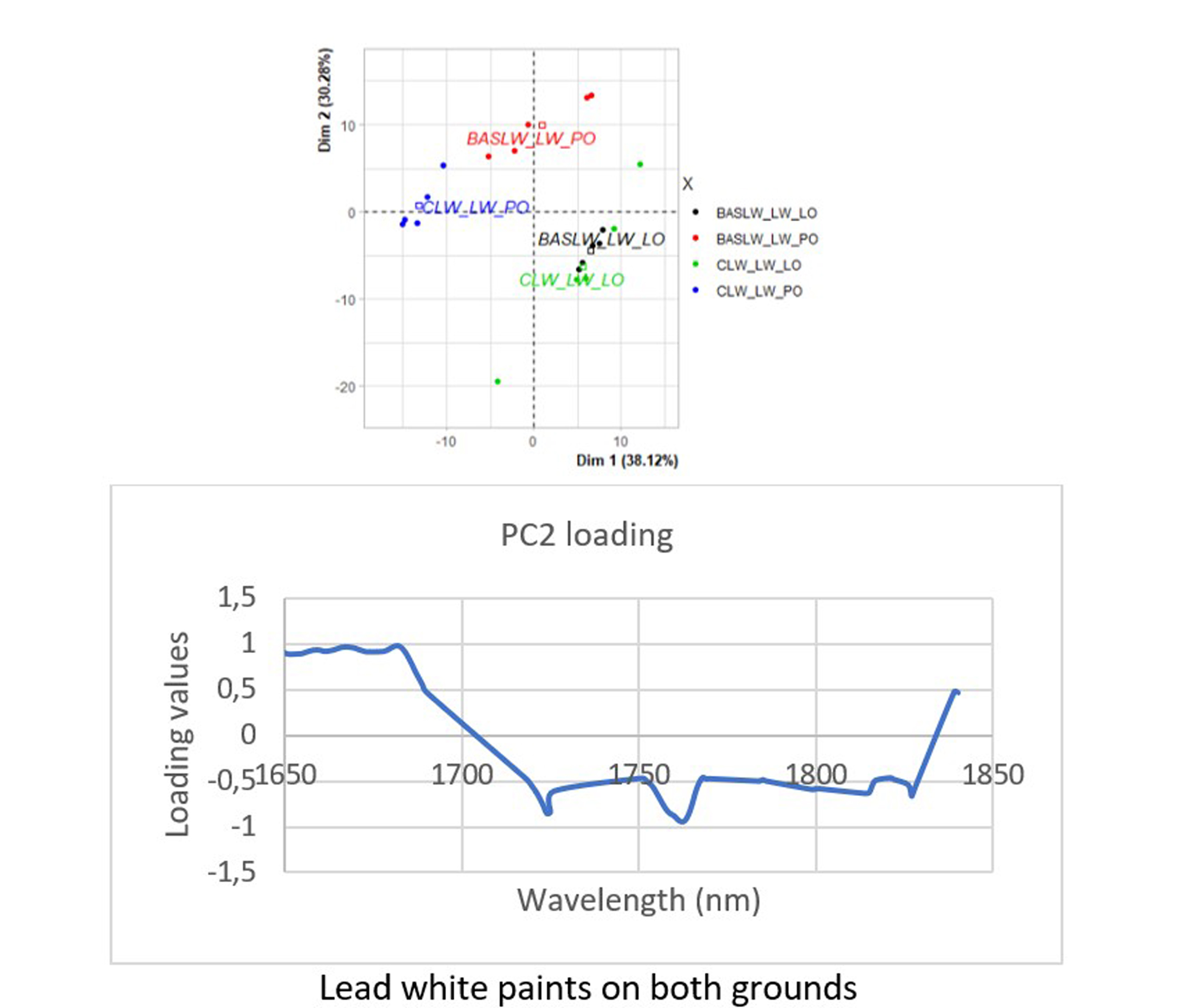

Supplement: Supplementary file 1 [file sensors-20-07125-s001.zip › Figure S2_2.jpg]

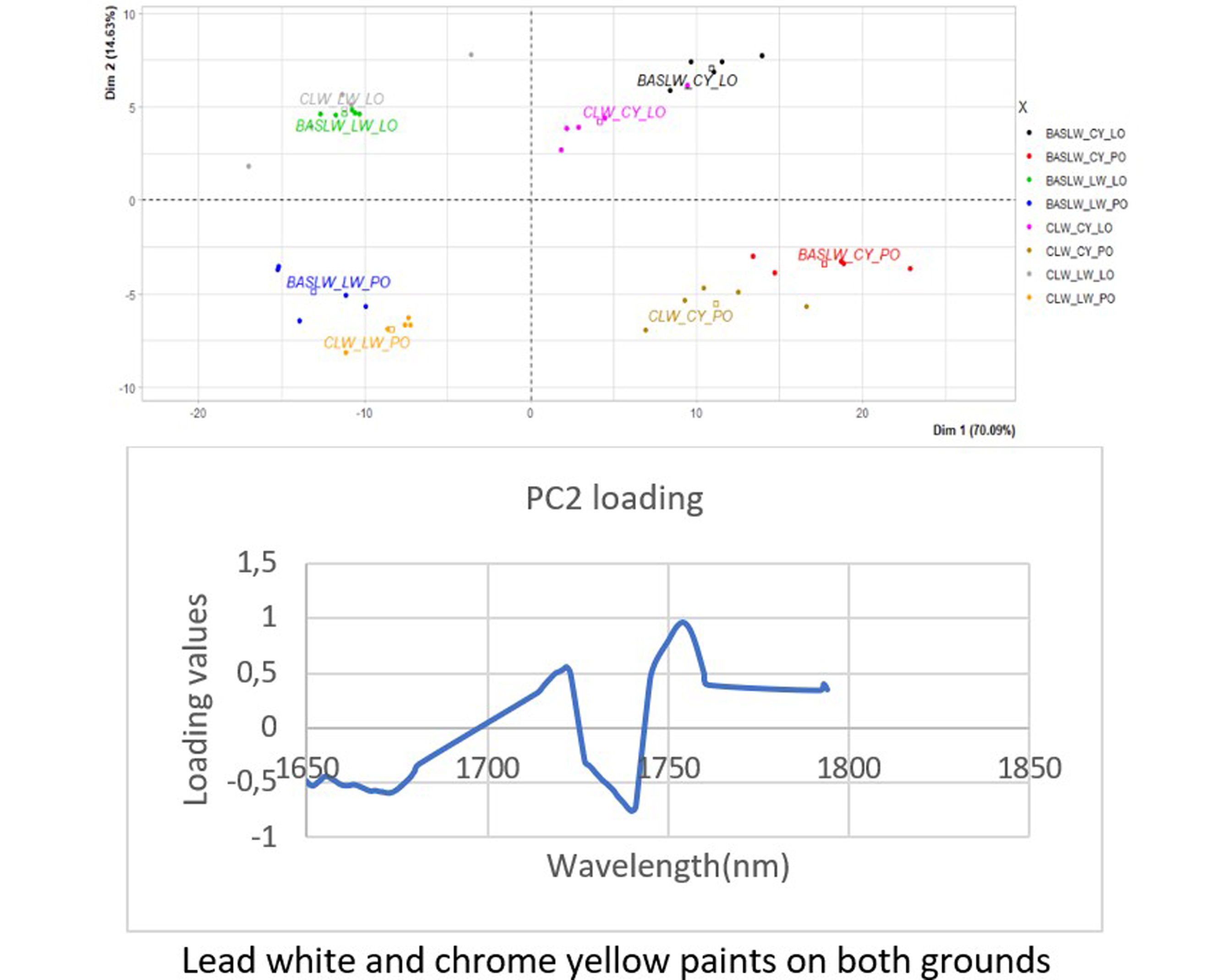

Supplement: Supplementary file 1 [file sensors-20-07125-s001.zip › Figure S2_3.jpg]

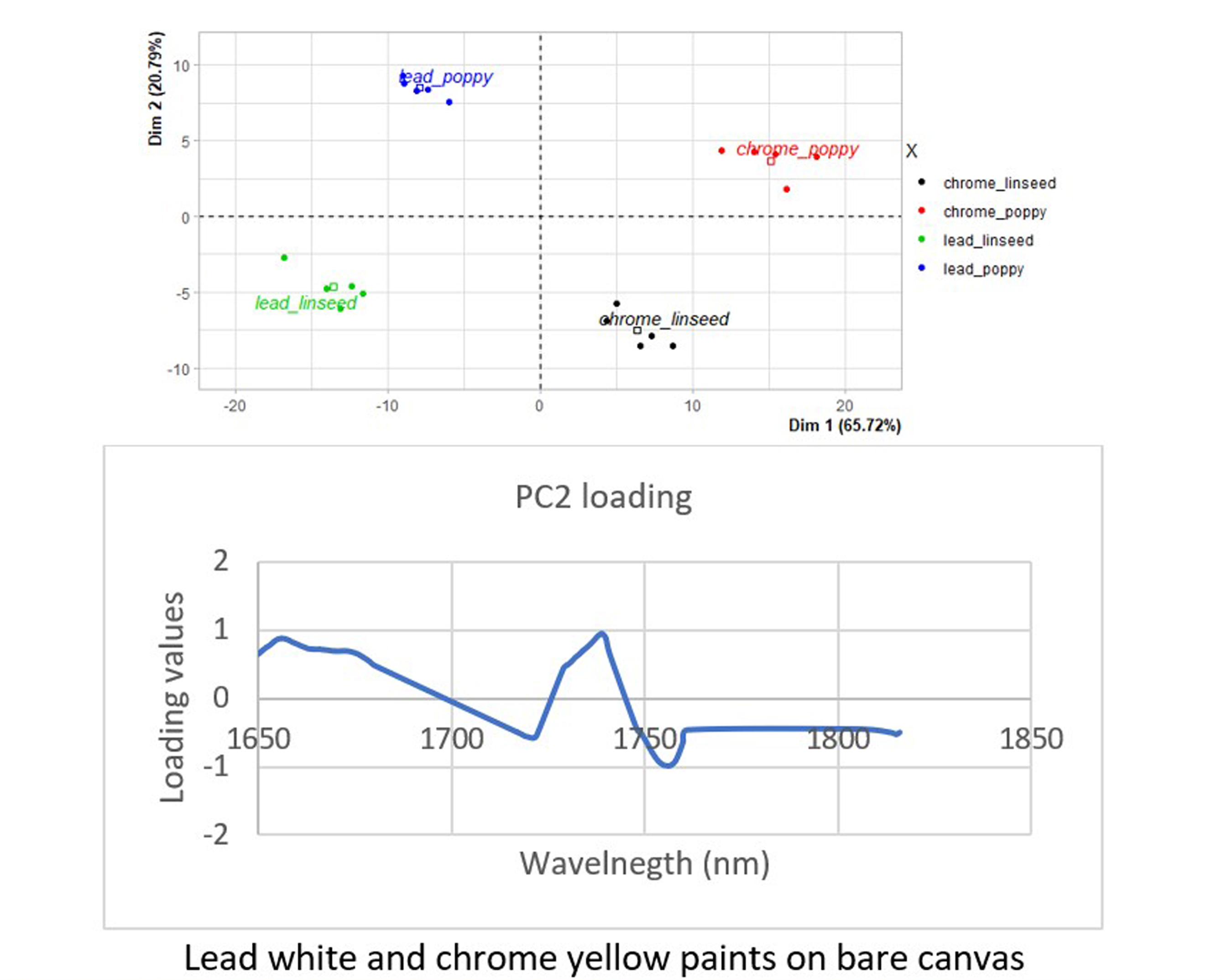

Supplement: Supplementary file 1 [file sensors-20-07125-s001.zip › Figure S2_4.jpg]

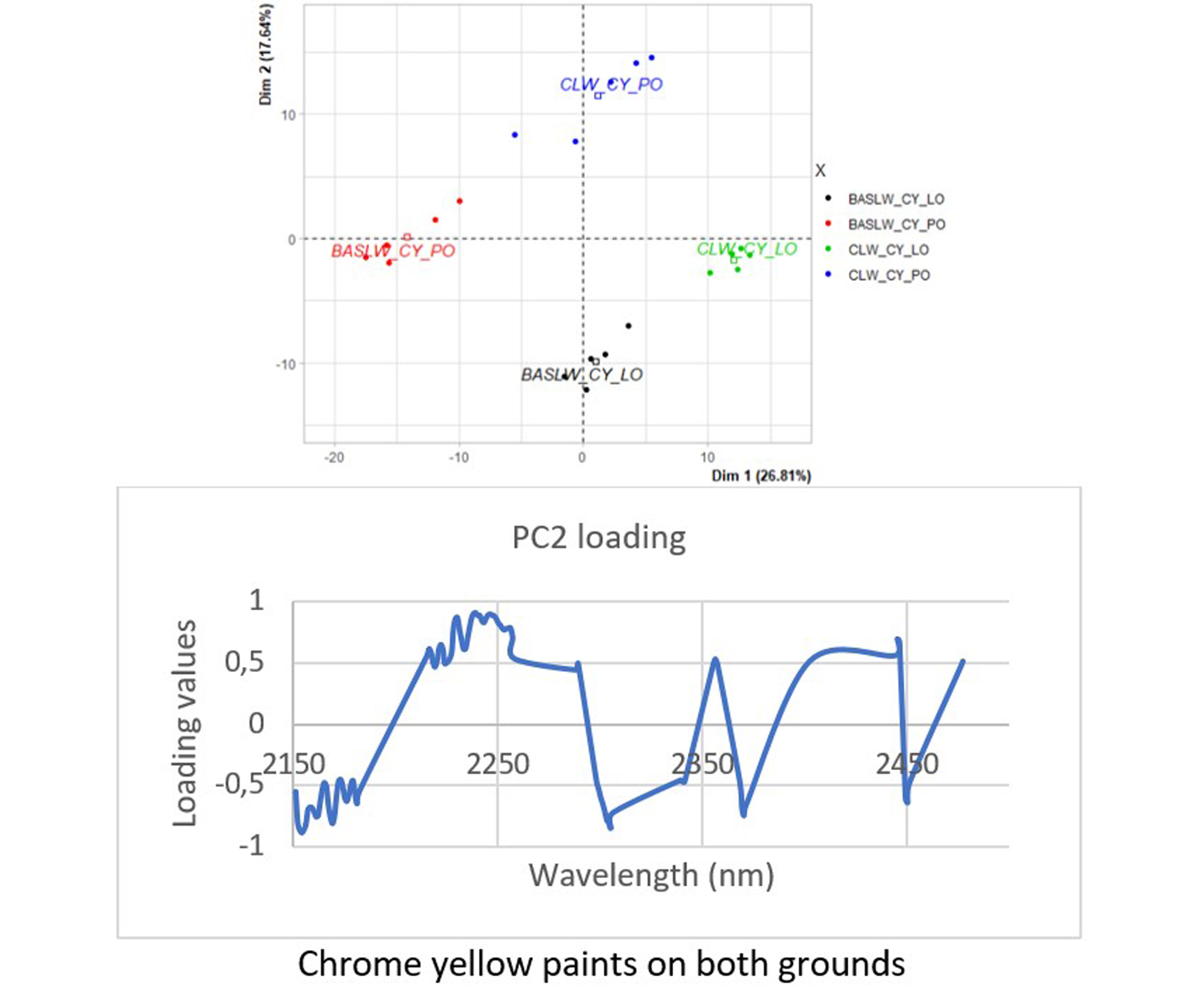

Supplement: Supplementary file 1 [file sensors-20-07125-s001.zip › Figure S3_1.jpg]

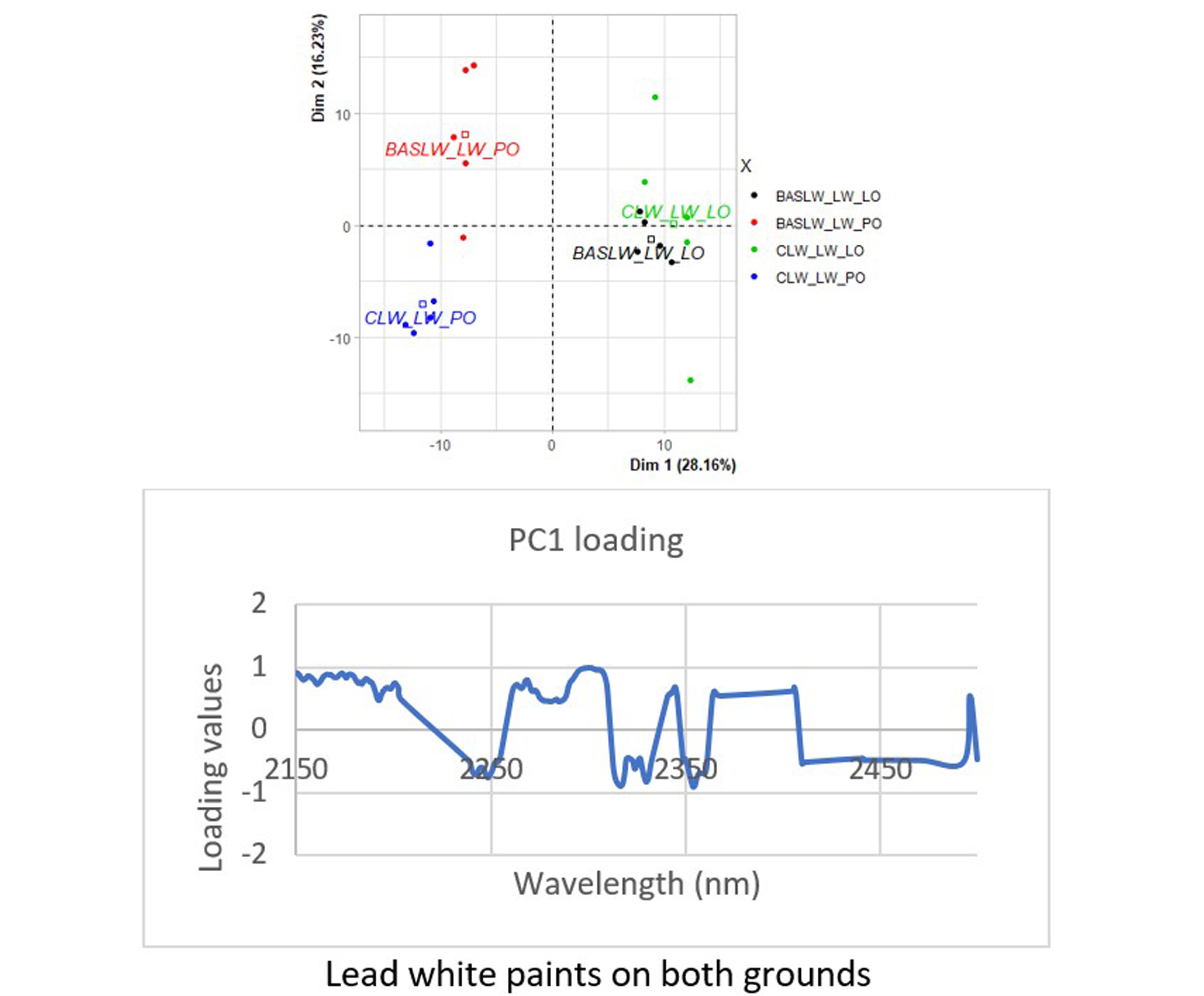

Supplement: Supplementary file 1 [file sensors-20-07125-s001.zip › Figure S3_2.jpg]

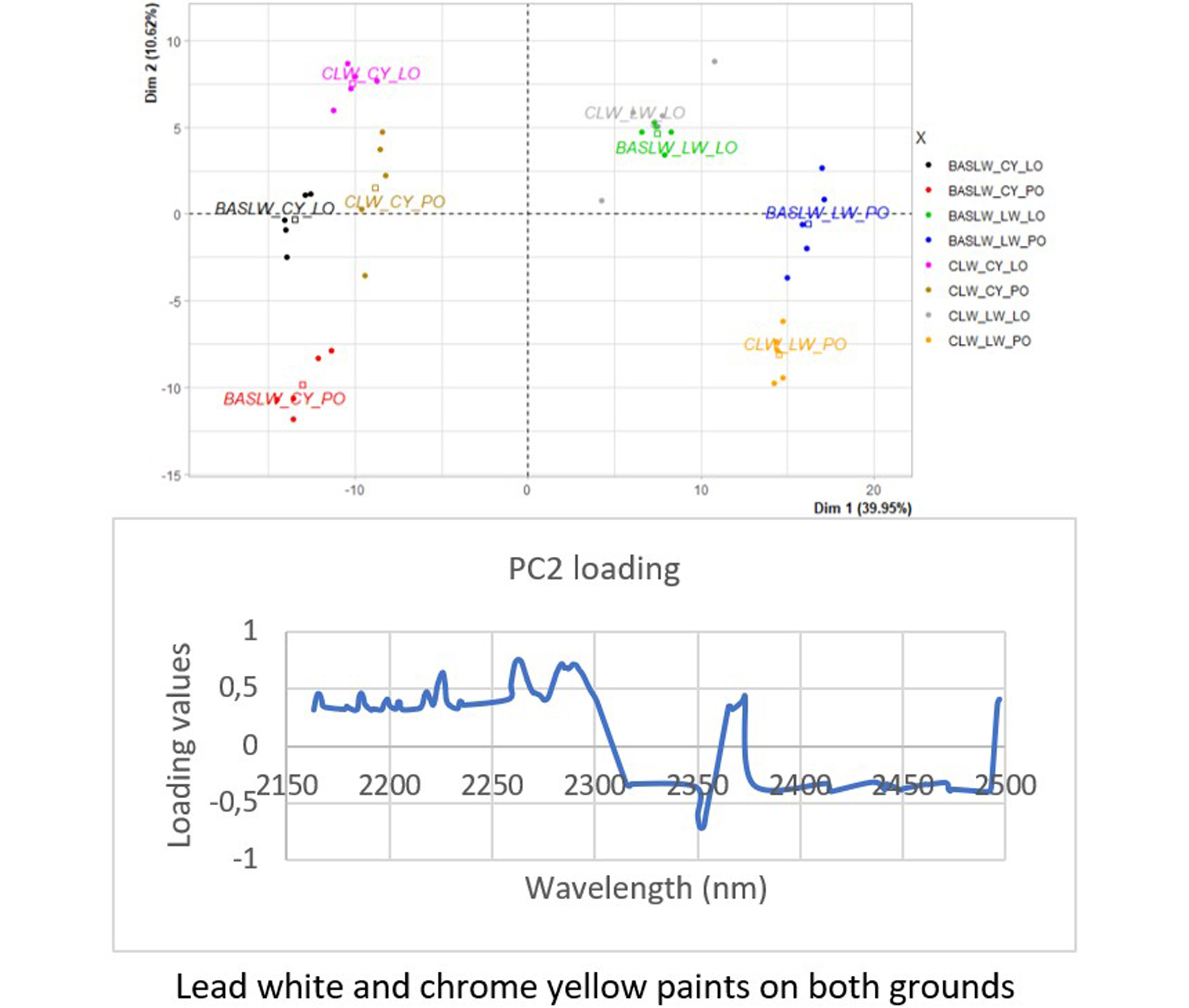

Supplement: Supplementary file 1 [file sensors-20-07125-s001.zip › Figure S3_3.jpg]

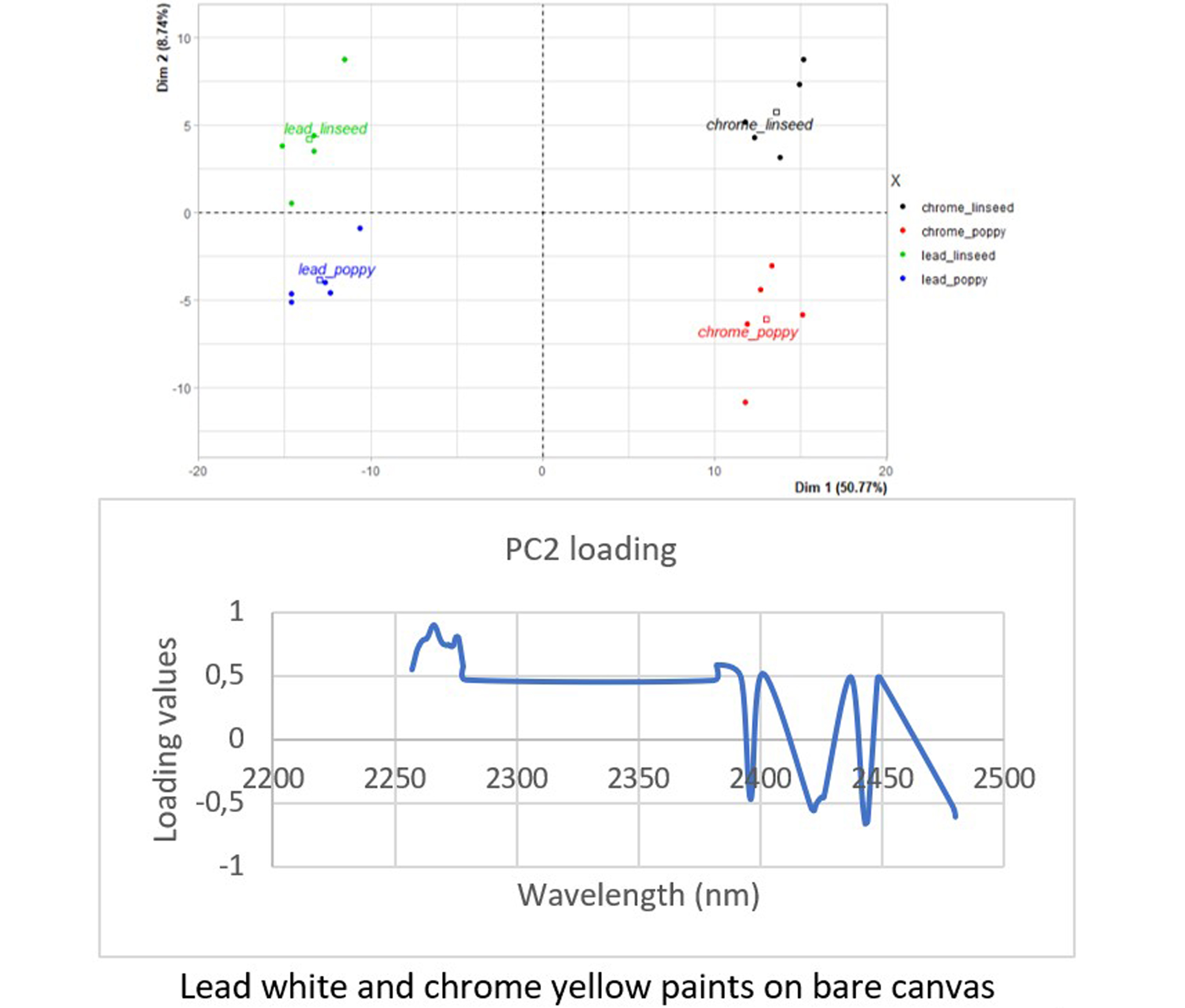

Supplement: Supplementary file 1 [file sensors-20-07125-s001.zip › Figure S3_4.jpg]

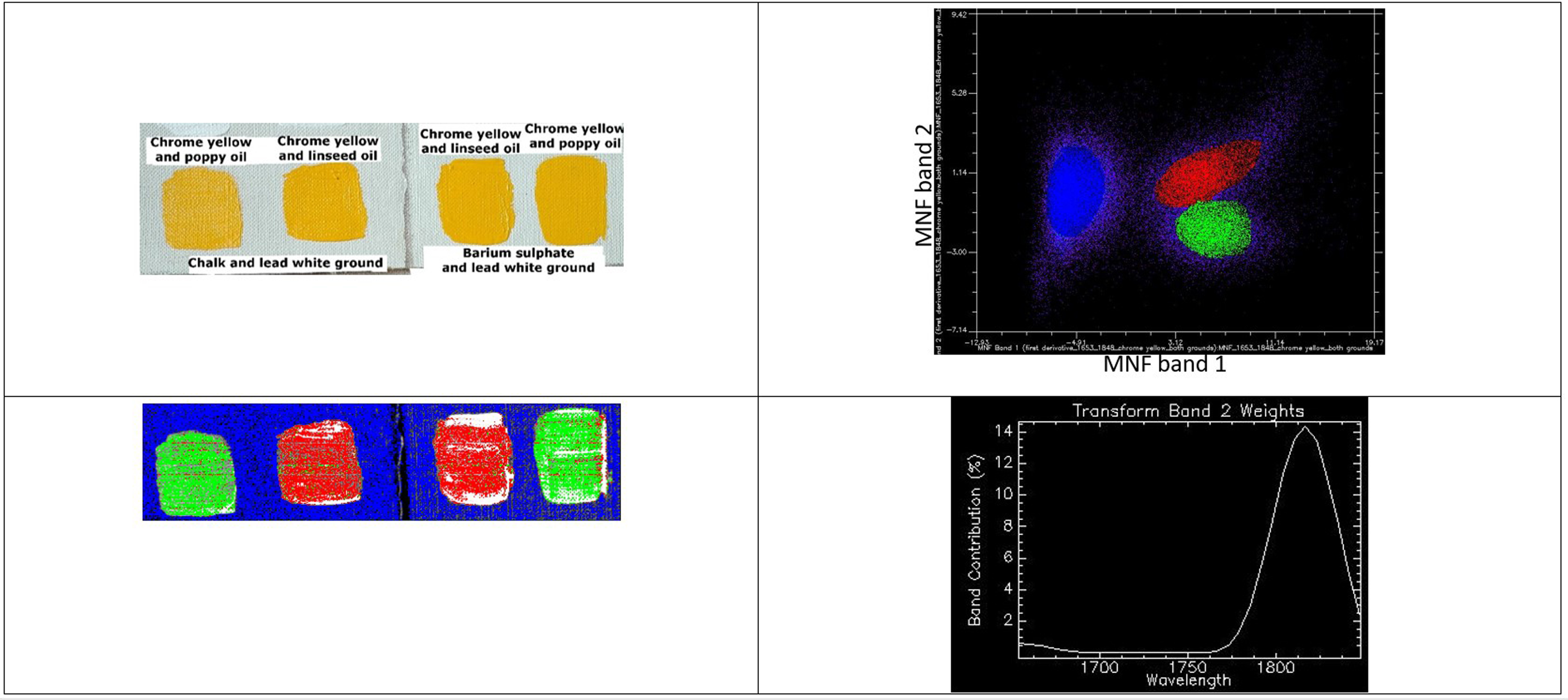

Supplement: Supplementary file 1 [file sensors-20-07125-s001.zip › Figure S4.jpg]

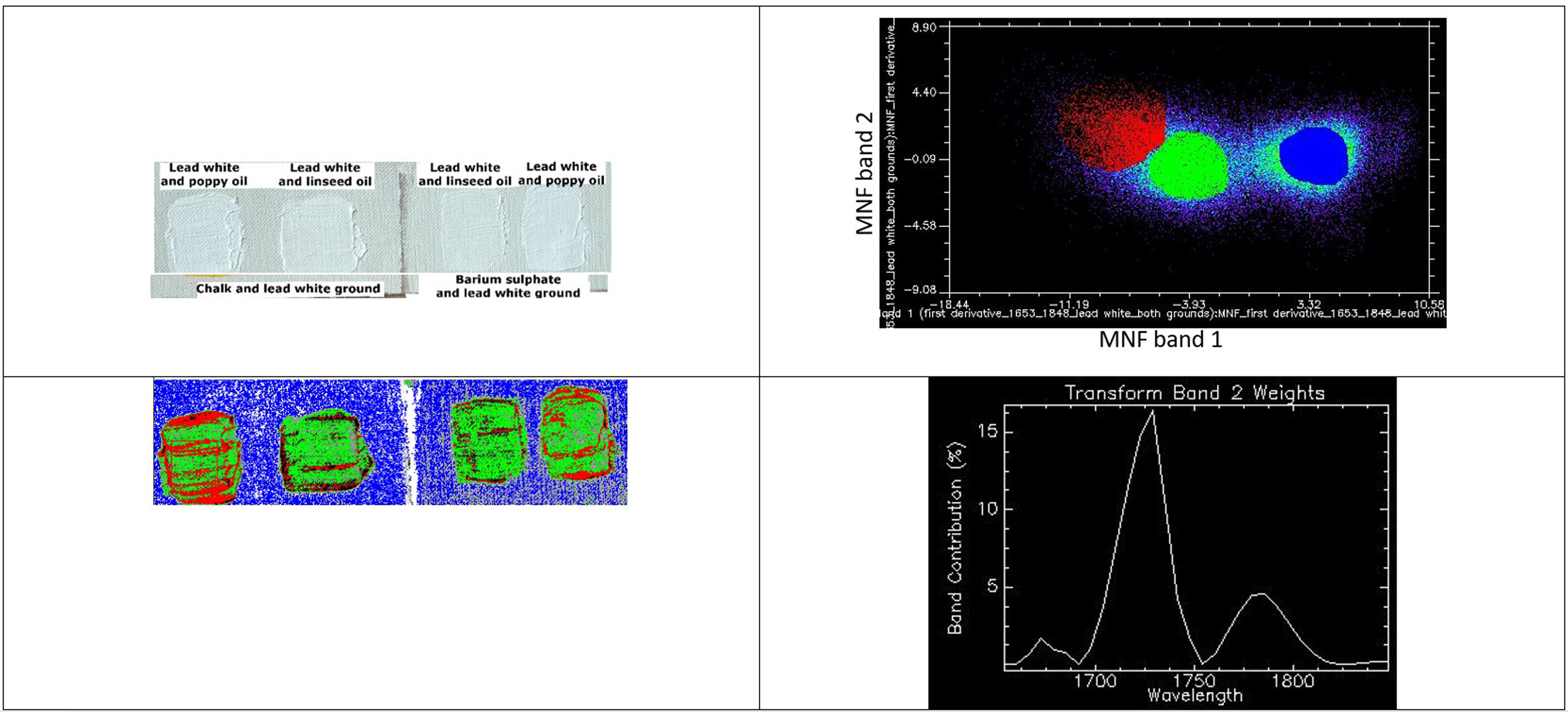

Supplement: Supplementary file 1 [file sensors-20-07125-s001.zip › Figure S5.jpg]

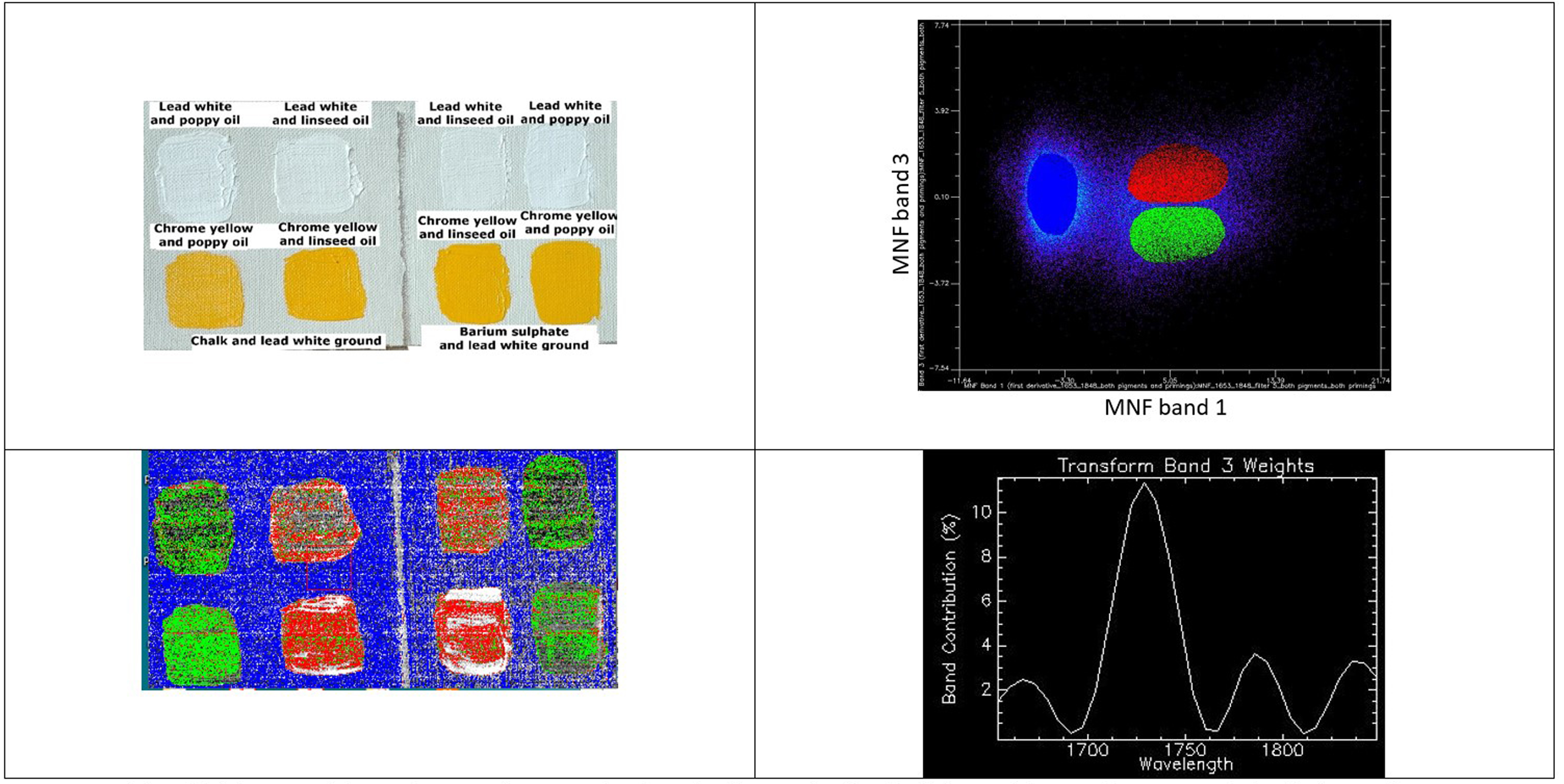

Supplement: Supplementary file 1 [file sensors-20-07125-s001.zip › Figure S6.jpg]

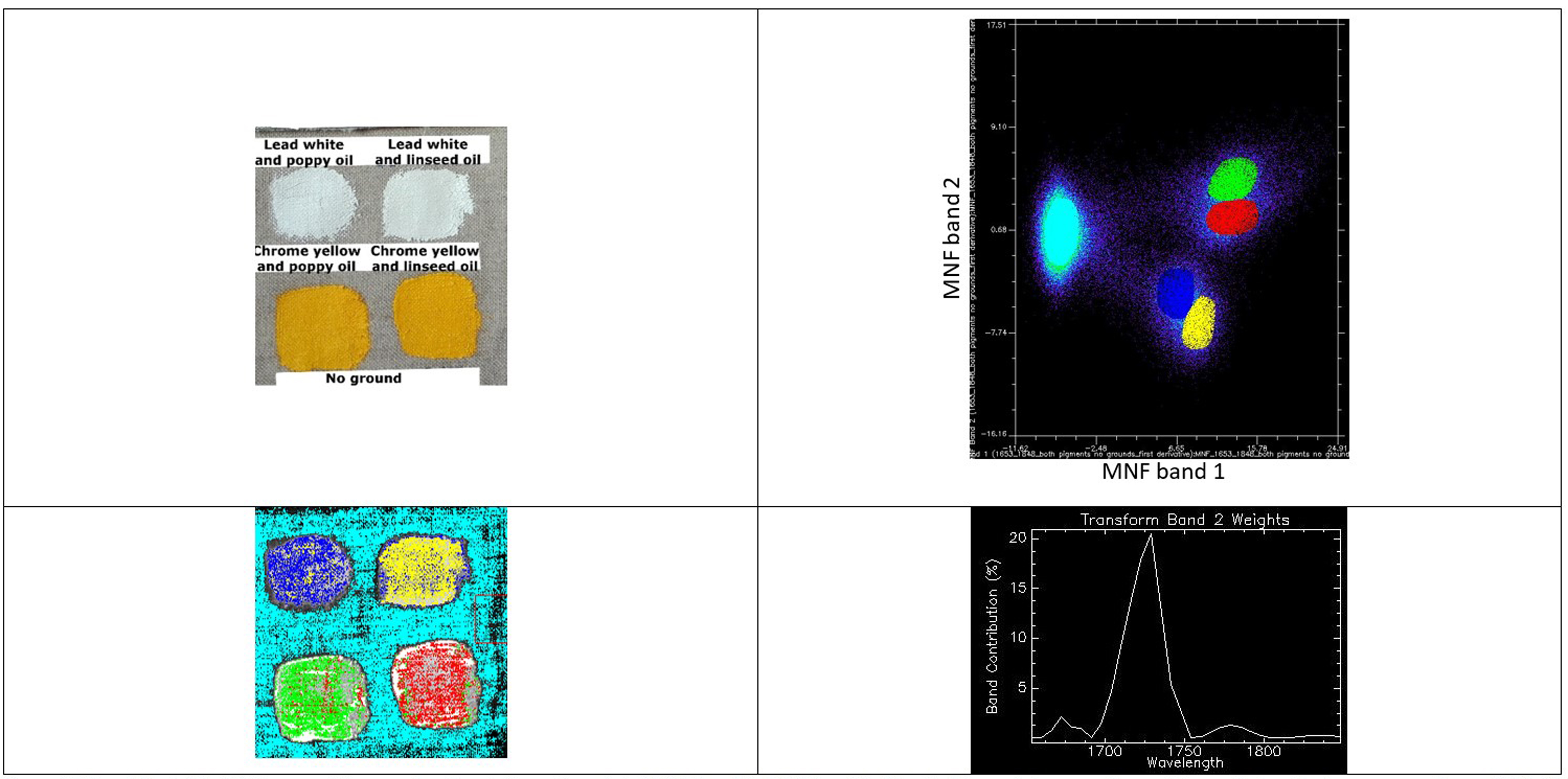

Supplement: Supplementary file 1 [file sensors-20-07125-s001.zip › Figure S7.jpg]
